# How hype and hyperbole distort the neuroscience of sex differences

**DOI:** 10.1371/journal.pbio.3001253

**Published:** 2021-05-10

**Authors:** Gina Rippon, Lise Eliot, Sarah Genon, Daphna Joel

**Affiliations:** 1 Aston Brain Centre, College of Health and Life Sciences, Aston University, Birmingham, United Kingdom; 2 Stanson Toshok Center for Brain Function and Repair, Chicago Medical School, Rosalind Franklin University of Medicine & Science, North Chicago, Illinois, United States of America; 3 Institute for Neuroscience and Medicine, Brain & Behavior (INM-7), Research Center Jülich, Jülich, Germany; 4 Institute for Systems Neuroscience, Medical Faculty, Heinrich-Heine University Düsseldorf, Düsseldorf, Germany; 5 School of Psychological Sciences, Tel-Aviv University, Tel-Aviv, Israel; 6 Sagol School of Neuroscience, Tel-Aviv University, Tel-Aviv, Israel; Oxford University, UNITED KINGDOM

## Abstract

Mind your language! This Perspective article points out that the source of misreporting in the field of sex/gender differences can often be found in the unjustified overstatements used by researchers themselves in reporting their findings; caution is needed when addressing differences between human beings, and hype and hyperbole should be avoided.

## Teaser

Sex/gender differences in the human brain attract attention far beyond the neuroscience community. Given the interest of nonspecialists, it is important that researchers studying human female–male brain difference assume greater responsibility for the accurate communication of their findings.

Research into female–male brain differences is believed important for understanding sex/gender disparities in neurological and mental health, in educational and occupational achievement, and for informing diversity and inclusion initiatives. (We use “sex/gender” to embrace the difficulty of disentangling participants’ “sex”—biological attributes including genitalia, sex-related chromosomes, and hormones—and “gender”—psychological and social attributes associated with males and females—as distinct variables in human neuroscience.) Interest in the outcomes of brain sex/gender difference research extends beyond the research specialty itself, calling for attention to issues of transparency and clarity in communicating such findings.

Concerned researchers have warned about the ease with which new and existing datasets can be mined for male–female group differences, often leading to reporting bias and false positives or failures to report effect sizes where differences have been found [1–3]. Likewise, they’ve raised concerns about the misuse of sex/gender brain findings in the public sphere, where the data have been translated for popular communication in careless and stereotypical ways [3,4]. Much less attention has been paid to problems of misrepresentation arising from the narrative and interpretive context in scientific articles themselves. Here, we highlight the need for “impression management” in research reports on brain sex/gender difference.

A recurrent problem in such studies is that the qualitative terminology used to describe the results does not accurately reflect the actual findings. Contemporary brain imaging research employs datasets with hundreds or thousands of measures, which are analyzed using multiple comparisons. Frequently, statistically significant differences are found in only a small fraction of possible contrasts. But this is rarely made clear in the abstract and discussion section, even if it is acknowledged in the results. For example, in a study of brain connectivity networks, the abstract states that sex differences were “prominent … at multiple scales of analysis” despite only 2% of the thousands of comparisons showing small statistical differences [5]. Such hyperbole can be compounded when there is unjustified emphasis on marginally significant findings and/or findings that did not actually survive correction for multiple comparisons. For example, the title of another recent paper referred to sex differences in “brain growth trajectories” even though none of the 46 critical measures showed significant sex-by-age differences after correcting for multiple comparisons [6]. The discussion further focused on sex/gender differences that had not survived appropriate statistical corrections.

The preference for positive results in scientific publications is an acknowledged problem [1,2,4], but is no excuse for glossing over the larger context of any statistically significant findings. To ensure an accurate reflection of all statistical comparisons, journal editors and reviewers should require that these be reported by indexing the number of (genuine) statistically significant differences to the total number of comparisons made or as a ratio of differences to similarities. This should be included in the abstract, results, and discussion sections to help readers gauge the true degree of group-level differences. Verbal summaries of the findings should use terms such as “many,” “strong,” etc. only when justified by this numerical index.

A second set of problems emerges when sex/gender comparisons are conducted by investigators naïve to the field or as an “add-on” to the main objective of the study. Such researchers commonly adopt an essentialist binary framework and an evolutionary perspective that biases the analysis, design, and interpretation of results. The underlying assumption is that female–male differences are determined by biological factors (i.e., “sex”), ignoring the myriad of psychosocial influences (i.e., “gender”) that can affect the brain and may not have been assayed as possible covariates or considered when interpreting the results. For example, a paper on “social brains” [7] interpreted some limited male-female differences in correlations between certain brain structures and social variables as evidence “that human survival has been optimized toward sex-specific strategies to successfully navigate the social world (7, p. 9).” Considering the interest of such research to nonexperts, journal editors and reviewers should ensure authors acknowledge the full biopsychosocial complexity of sex/gender and avoid the impression that there is a single, well-established, and noncontroversial interpretation of their findings.

This leads us to the third issue: the ease of deriving a post hoc rationalization for discovery-based findings of sex/gender brain differences. The core of this problem is a failure to acknowledge that the link between structure and function in the human brain is not well defined. Most mental processes engage many overlapping neural structures and circuits, so researchers have a wide range of choices in the behavioral interpretation they apply to any sex/gender difference in structure or connectivity. This makes it all too easy to retroactively spin a speculative relationship to some gender disparity around any differences detected in large-scale human neuroimaging databases, such as a high-profile article that interpreted modest connectome sex/gender differences as supporting “co-ordinated action” by male brains versus “communication” by female brains [8]. Although an important solution for this issue is the preregistration of research protocols, where post hoc analyses are regulated and selective inference is detected [9], impression management in the final communication of research findings can remain a problem.

Editorial policy and reviewer guidelines commonly focus on methodological issues and pay less attention to the responsible use of language in the final text, even though the title and abstract are often the sole source of a study’s take-home message for nonspecialists.

In the interests of both science and society, neuroscientists need to think carefully about how they present findings about brain differences between socially segregated groups of healthy humans. They need to recognize that any neurobiological comparison between such groups raises the potential for stereotyping and stigmatization. That means ensuring that research design and methodology reflect current understanding of sex/gender. It also means paying careful attention to the impression given by selective narrative framing and inaccurate use of quantitative descriptors. A failure to clarify the practical significance of complex statistical findings or to acknowledge the multifaceted biopsychosocial contributions to sex/gender groupings gives undue weight to the relevance of the findings. Given the real costs of entrenched sex/gender disparities across society, neuroscientists have a duty to prevent the spread of misinformation about the neural basis of such differences.
